# Supplementary material for: A cost- and time-efficient method for high-throughput cryoprocessing and tissue analysis using multiplexed tissue molds
Source: Cell Rep Methods. 2025 Jun 27;5(4):101023. doi: 10.1016/j.crmeth.2025.101023 (PMC12256940; doi:10.1016/j.crmeth.2025.101023)
Supplement: Document S1. Figures S1–S4 and Table S1 [file mmc1.pdf]

Cell Reports Methods, Volume *5*

Supplemental information

A cost- and time-efficient method for high-throughput cryoprocessing and tissue analysis using multiplexed tissue molds

Daniel Reumann, Martin Colombini, Paul Möseneder, Agnieszka Piszczek, and Jürgen A. Knoblich

A

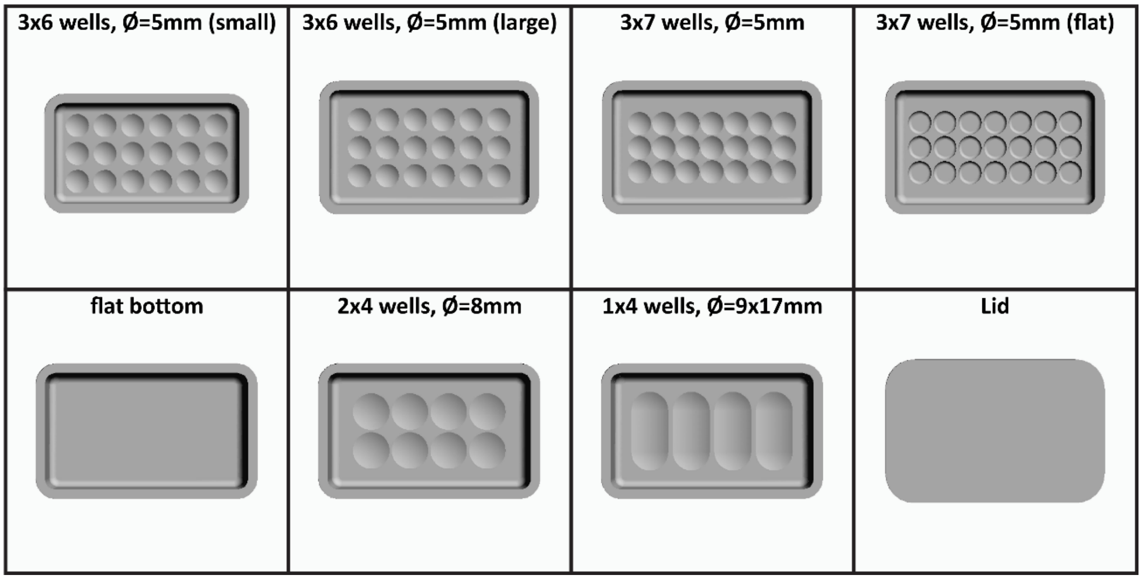

Supplemental figure 1: MTM designs. Related to figures 1-3.

A, MTM designs for various tissue sizes and numbers, adjusted for regular size microscopy slides, as well as the lid design.

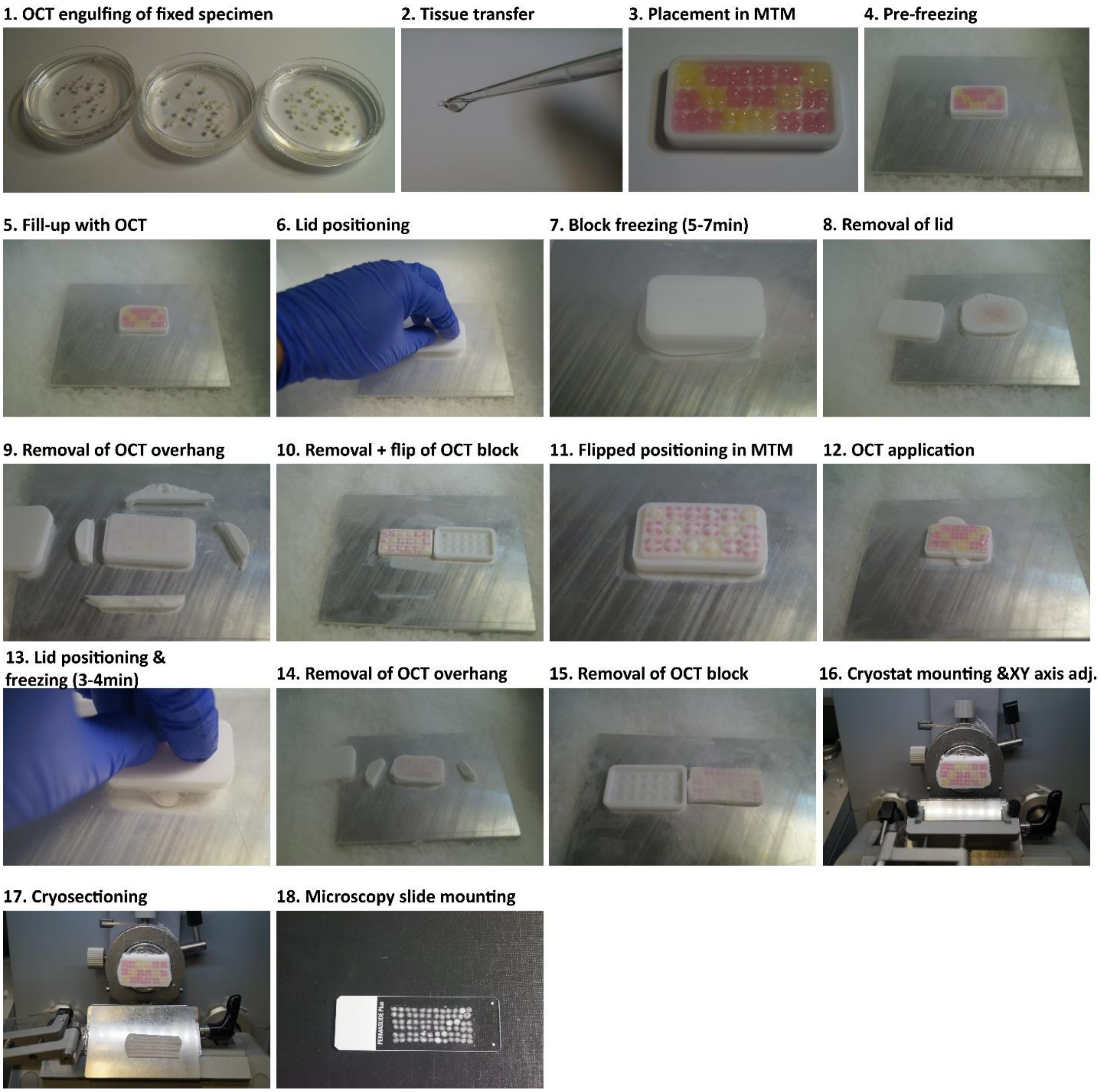

Supplemental Figure 2: Step-by-step documentation of MTM based multiplexed cerebral organoid embedding and cryosectioning. Related to figures 1-3.

Organoids expressed either GFP, tdtomato or no fluorophore (see Figure 1C, step 6).

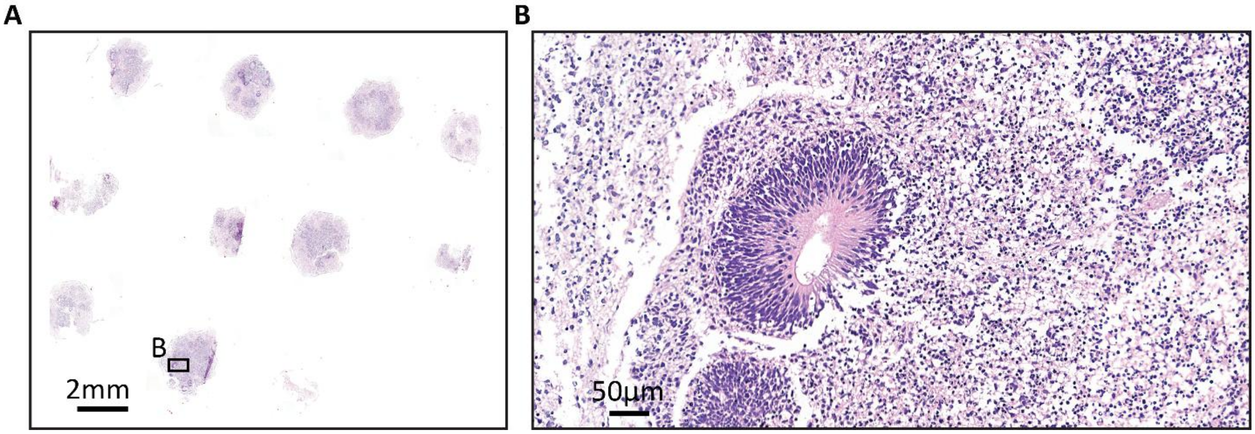

Supplemental figure 3: Paraffin embedding using MTMs. Related to Figure 1.

A, Overview of microtome sectioned organoids which were dehydrated, and paraffin embedded in MTMs (4x4 organoids with last position empty) and then sectioned on a vibratome and H&E stained. **B**, magnified view of indicated box in panel A.

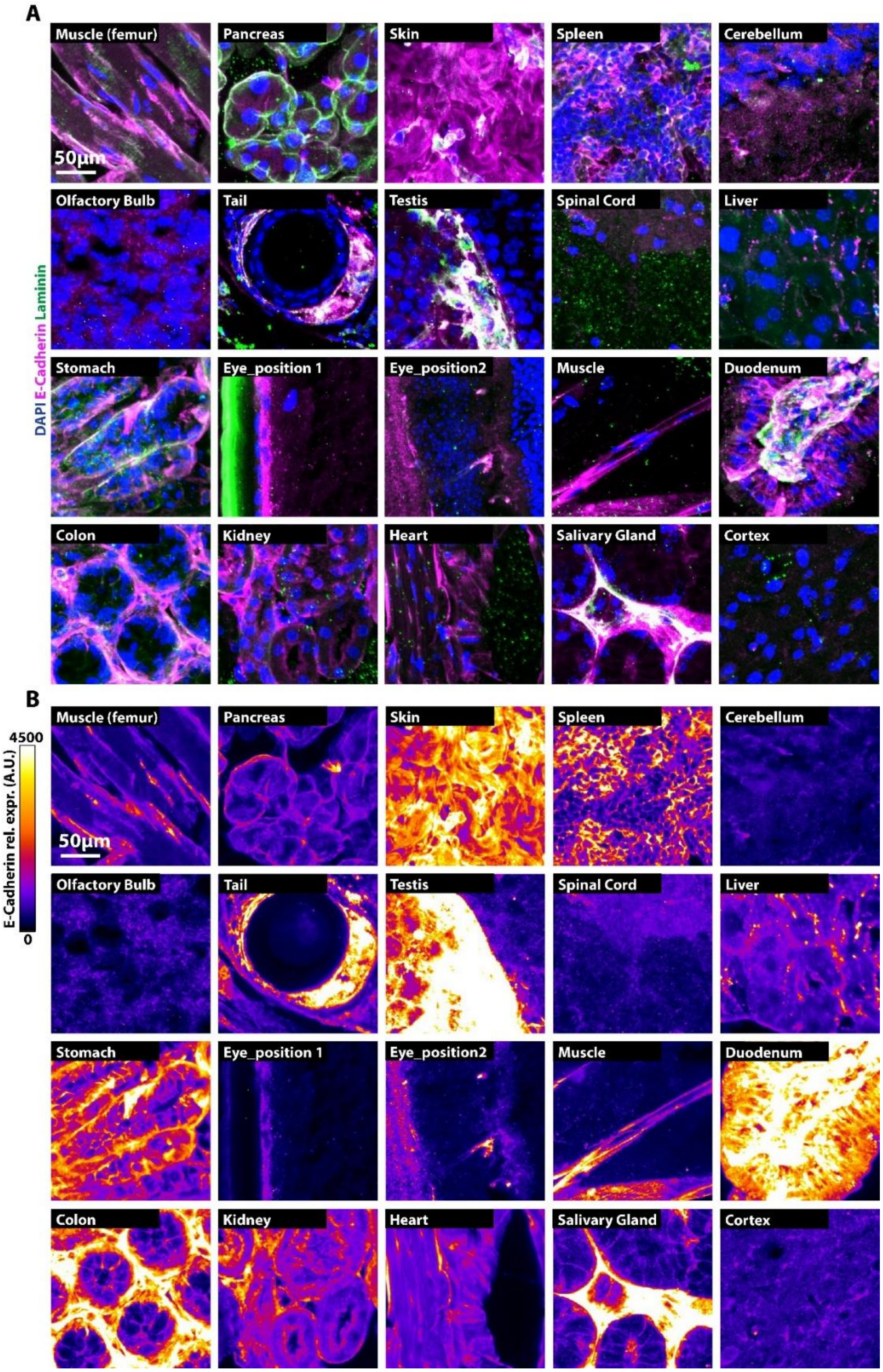

Supplemental Figure 4: High-Magnification recordings of MTM processed mouse tissues. Related to figure 2.

A, Representative 40x immunofluorescence images of MTM processed mouse organs (see also Figure 2) labeled for DAPI, E-Cadherin and Laminin. **B,** Corresponding Fire LUT representation highlighting tissue-specific E-cadherin expression levels. N= 1 mouse and 19 organs.

Supplemental Table S1: Cost list for timecourse organoid experiment. Related to Figures 1 and 3.

General Consumables			Product links	Cat No	Accessed	Price
TX100 50g (Thermo)	1.916	€/ml	https://www.thermofisher.com/order/catalog/product/85111	28313	accessed 07/01/2024	1l for 1916€
BSA (AlbuMAX) 11021037 (Thermo)	4.05	€/g	https://www.thermofisher.com/order/catalog/product/11021037?SID=srch-srp-11021037	11021037	accessed 07/01/2024	100g for 405€
PBS sterile thermo 1l 10010031	0.03282	€/ml	https://www.thermofisher.com/order/catalog/product/10010031?SID=srch-srp-10010031	10010031	accessed 07/01/2024	1l for 32,82
PBS tablets nonsterile 100 tablets (500ml each)	194	€	https://www.thermofisher.com/order/catalog/product/18912014	18912014	accessed 07/01/2024	100 tablets for 194€ (500ml each)
-->PBS tablet derived PBS	0.00388	€/ml				

Cost estimate- OCT			https://www.scienceservices.eu/tissue-tek-oct-compound.html	SA62550-12	accessed 07/01/2024	12 bottles a 125ml for 214,90€
OCT (12 bottles)	214.9	€				
per block (approx 10ml OCT):	1.517655	€				
6 wells of 6well plate (reusable), approx 24ml:	3.642373	€				
**total**	**5.160028**	€				

Cost estimate for 50ml antibody staining solution		
TX100	0.0958	€
BSA	10.125	€
PBS	0.194	€
**SUM**	**10.4148**	**€**
**SUM for 10 slides (approx. 5ml)**	**1.04148**	**€**

Cost estimate for 50ml blot/perm solution		
TX100	0.2874	€
BSA	10.125	€
PBS	0.194	€
**SUM 50ml**	**10.6064**	**€**
**SUM for 10 slides (approx. 5ml)**	**1.06064**	**€**

Cost estimate 1l PBS-T		
PBS tablet	3.88	€
TX100	0.1916	€
**SUM**	**4.0716**	€

1l PBS		
2PBS tablets (e.g. Thermo 18912014)	3.88	€
**SUM**	**3.88**	**€**

Cost estimate for 10 primary antibody IHC (200ul/staining)		
approximation:		
cost for 100µl antibody (estimated dilution: 1:300):	500	€
--> antibody costs per slide (0.67µl in 200µl AB solution):	3.35	€
**SUM (antibody cost for 10 stainings):**	**33.5**	**€**

Cost estimate for 10 secondary antibodies (fluorescently conj., 200µl/staining)		
approximation:		
per antibody for 500ul (2mg/ml conc, est. dil. 1:500):	363	€
--> antibody cost per slide (0.4µl in 200µl):	0.2904	€
**SUM (secondary antibody cost for 10 slides)**	**2.904**	**€**

Microscopy slides			Product links	Cat No	Accessed	Price
VWR 631-0108 72 slides	36.8	€	https://at.vwr.com/store/product?keyword=631-0108	631-0108	accessed 07/01/2024	36.8€ for 72 slides
**SUM (10 microscopy slides total)**	**5.111111**	**€**				

Coverslips			Product links	Cat No	Accessed	Price
VWR 24x50 631-0146 1000 pieces	152	€	https://at.vwr.com/store/product?keyword=%20631-0146	631-0146	accessed 07/01/2024	152€ for 1000 coverslips
**SUM (10 coverslips)**	**1.52**	**€**				

Mounting media			Product links	Cat No	Accessed	Price
e.g. DAKO 15ml	110	€	https://www.agilent.com/store/productDetail.jsp?catalogId=S302380-2&catId=SubCat3ECS_866	CS70330-2	accessed 07/01/2024	110€ for 15ml
SUM (for 10 slides, approx. 30µl/slide)	2.2	€				

TOTAL COST (MTM):		
**TOTAL CONSUMABLE COST (1 antibody/slide):**	**60.4489**	€
**TOTAL CONSUMABLE COST (3 antibodies/slide):**	**47.4893**	€

Cost per Analysis (approx. 114 tissues per slide (dependent on position in the block), 21 tissue groups):		
Cost per tissue (for 10 stainings, 1 staining per slide):	0.530253	€
Cost per tissue group (for 10 stainings, 1 staining/slide):	2.878517	€
Cost per tissue (for 10 stainings, 3 staining per slide):	0.416573	€
Cost per tissue group (for 10 stainings, 3 staining/slide):	2.261396	€

TOTAL COST (conventional cryomolds):			Product links	Cat No	Accessed	Price
21 groups processed separately, 10 slides for AB staining:	210	slides				
multiplexed (3 antibodies/slide, 4 slides per group):	84	slides				
Single-use cryomolds (Tissue-Tek 15x15x15, 100pc):	37.9	€	https://www.scienceservices.de/tissue-tekr-cryomoldr-kryoeinbettformen.html	SA62534-10	accessed 07/01/2024	37.9€ for 100 pc
**Total consumable cost (1 antibody/slide):**	**1349.016**	**€**				
**Total consumable cost (3 antibody/slide, 4 slides):**	**1076.866**	**€**				

Cost saving with MTM:		
**1 antibody/slide:**	**95.519**	**%**
**3 antibodies/slide, 4 slides:**	**95.59**	**%**
