# Supplementary material for: A cost- and time-efficient method for high-throughput cryoprocessing and tissue analysis using multiplexed tissue molds
Source: Cell Rep Methods. 2025 Jun 27;5(4):101023. doi: 10.1016/j.crmeth.2025.101023 (PMC12256940; doi:10.1016/j.crmeth.2025.101023)
Supplement: Document S2. Article plus supplemental information [file mmc3.pdf]

Cell Reports
Methods

Report

A cost- and time-efficient method for high-throughput cryoprocessing and tissue analysis using multiplexed tissue molds

Graphical abstract

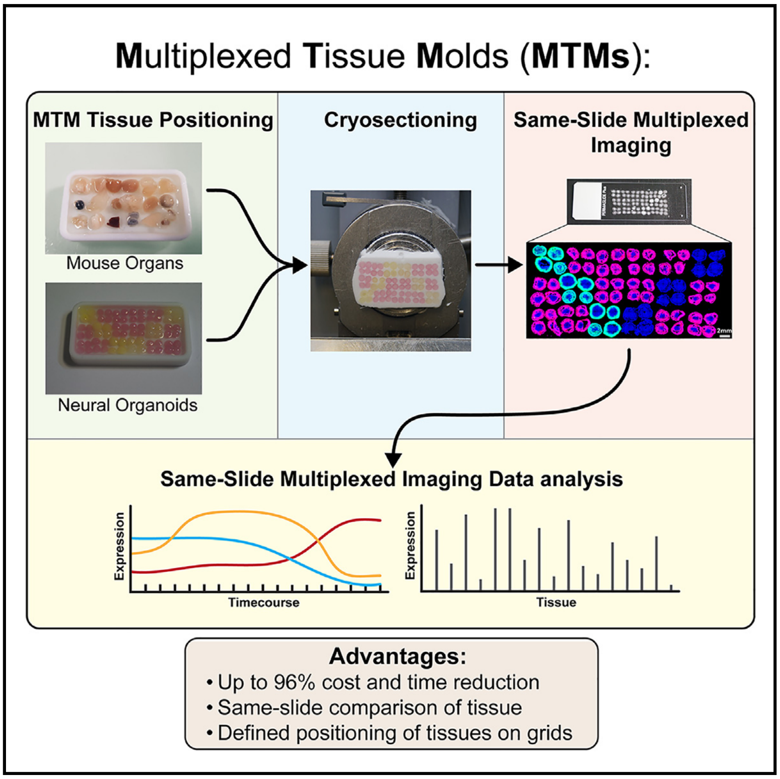

Highlights

- Multiplexed tissue molds (MTMs) drastically upscale cryosectioning procedures
- MTMs can simultaneously accommodate up to 19 mouse organs and ~110 cerebral organoids
- MTMs reduce analysis costs and processing times of tissues by up to 96%
- MTMs could be used to reduce diagnostic costs and for spatial transcriptomics

Authors

Daniel Reumann, Martin Colombini, Paul Möseneder, Agnieszka Piszczek, Jürgen A. Knoblich

Correspondence

daniel.reumann@dpag.ox.ac.uk (D.R.),
juergen.knoblich@imba.oeaw.ac.at (J.A.K.)

In brief

Reumann et al. develop multiplexed tissue molds (MTMs), which allow upscaling of tissue processing (up to 19 mouse organs or ~110 cerebral organoids simultaneously) while reducing workload and associated analysis costs by up to 96%. MTMs allow cryosection-based tissue analysis when labor, time, and cost are limiting factors and could be used for patient sample analysis as well as spatial transcriptomics approaches.

Check for updates

Reumann et al., 2025, Cell Reports Methods 5, 101023
April 21, 2025 © 2025 The Authors. Published by Elsevier Inc.
https://doi.org/10.1016/j.crmeth.2025.101023

CellPress

Cell Reports Methods

CellPress OPEN ACCESS

Report

A cost- and time-efficient method for high-throughput cryoprocessing and tissue analysis using multiplexed tissue molds

Daniel Reumann,[1,2,6,*] Martin Colombini,[3] Paul Möseneder,[1] Agnieszka Piszczek,[4] and Jürgen A. Knoblich[1,5,7,*]

[1]Institute of Molecular Biotechnology (IMBA) of the Austrian Academy of Sciences, Vienna BioCenter, Vienna, Austria
[2]Vienna BioCenter, Doctoral School of the University of Vienna and Medical University of Vienna, Vienna, Austria
[3]Institute of Molecular Pathology (IMP), Vienna BioCenter, Vienna, Austria
[4]Vienna BioCenter Core Facilities (VBCF), Vienna BioCenter, Vienna, Austria
[5]Department of Neurology, Medical University of Vienna, Vienna, Austria
[6]Present address: Kavli Institute of Nanoscience Discovery, University of Oxford, Oxford, UK
[7]Lead contact
*Correspondence: daniel.reumann@dpag.ox.ac.uk (D.R.), juergen.knoblich@imba.oeaw.ac.at (J.A.K.)
https://doi.org/10.1016/j.crmeth.2025.101023

MOTIVATION Efficient cryosectioning remains a critical yet labor- and cost-intensive step for immunohistochemistry and *in situ* hybridization, limiting routine diagnostic and research applications. The increasing demand for high-throughput tissue analysis—driven by advances in organoid and three-dimensional (3D) culture systems and tissue analysis for diagnostics—necessitates methods capable of processing numerous heterogeneous samples simultaneously. Current protocols lack multiplexing capabilities, leading to variability and extended processing times. Our work introduces multiplexed tissue molds (MTMs), a scalable solution that drastically reduces costs and labor by up to 96% while maintaining tissue integrity and consistency, thereby enabling large-scale (>100 tissues) comparative analyses and enhanced experimental reproducibility as well as access to tissue analysis, where cost is a restrictive factor.

SUMMARY

Cryosectioning remains the gold standard for antibody and transcriptomic/*in situ* hybridization tissue analysis. However, tissue processing is time-consuming and costly, limiting routine and diagnostic use. Currently, no commercially available protocols or products exist for multiplexing this process. Here, we introduce multiplexed tissue molds (MTMs) that enable high-throughput cryoprocessing—cutting costs and workload by up to 96% while permitting the processing of tissues of various sizes and origins. We demonstrate compatibility with heterogeneous tissues by processing 19 different adult mouse tissues in parallel. Furthermore, we process up to ∼110 neural organoids of different ages and sizes simultaneously and assess their neural differentiation marker expression. MTMs allow sectioning-based tissue analysis when labor, time, and cost are limiting factors. MTMs could be used to compare high specimen numbers in histopathological settings, organism-wide antigen and antibody targeting studies, high-throughput tissue screens, and defined tissue section positioning for, e.g., spatial transcriptomics experiments.

INTRODUCTION

While omics approaches are a huge step in the direction of holistic tissue characterization, cryosectioning-based immunohistochemical analysis is still the gold standard when it comes to the analysis of morphological aspects of tissues and cells, as well as protein and RNA expression analysis.[1,2] Despite the importance of immunohistochemistry (IHC), a key limitation of immunohistological analysis is the time-consuming specimen processing and costs associated with target labeling, and no commonly available system exists for multiplexing cryosectioning of tissues. Such systems would be of particular importance in fields where multiple tissues—or tissue groups—need to be compared to each other or where economic factors hinder the establishment of IHC as a routine application.

With the rise of organoid and three-dimensional (3D) tissue culture technologies, serial section analysis and comparisons across large sample numbers become increasingly important.[3] Such comparisons would ideally be performed in a single experiment, as even minor steps, such as antibody labeling on multiple

© 2025 The Authors. Published by Elsevier Inc.
This is an open access article under the CC BY license (http://creativecommons.org/licenses/by/4.0/).

slides, can cause heterogeneous labeling when performed on multiple slides. 3D IHC and tissue clearing have become powerful tools for 3D tissue analysis but remain relatively low throughput, have limitations in the number of antibodies per tissue, and require advanced microscopy and data storage and processing equipment.[4]

Here, we present a streamlined cryoprocessing method using reusable, custom-designed multiplexed tissue molds (MTMs) that enable the serial processing and staining of multiple tissue groups at once, tremendously reducing tissue processing time. Our technology allows direct comparison of antibody labeling by removing slide-to-slide variability and drastically reduces costs associated with labeling and analysis. We demonstrate the use of this method using MTM variants, which have the capacity for 4–21 specimens (or specimen groups) and easily fit on regular microscopy adhesion glass slides (75 × 25 mm) by processing up to 72 specimens (4 specimens per position in a 6 × 3 MTM) simultaneously.

We further demonstrate the wide applicability of this method in both the simultaneous processing of 19 different mouse tissue samples, proving that MTMs can be used for tissue sections ranging from soft brain to (decalcified) bone, and a time course experiment for protein expression during the early stages of neurogenesis in ~110 differently sized cerebral organoids, demonstrating the versatility of this method in differently sized tissues.

This method can cut tissue processing and labeling costs by up to 96% in comparison to serial tissue processing while also drastically reducing processing and analysis time, with the potential for even further savings enabled by scaled-up versions of these designs.

RESULTS

High-throughput cryosectioning: Design and implementation of MTMs

Commercially available embedding systems for cryoprocessing focus on either individual tissue group blocks (Figure 1A) or biopsy punch embedding ("tissue (micro)arrays")[5] and are limited in their potential for parallel processing. While tissue arrays allow the embedding of many tissues simultaneously, they have the limitation that the tissues must then be repeatedly thawed during cryosectioning to allow for the optimal cutting temperature (OCT) and tissue to attach—which can cause considerable damage to the tissue. Furthermore, tissue arrays do not allow for modified shapes and sizes of tissue but are limited to the selected size of a biopsy puncher. We aimed to develop a method for simultaneously embedding multiple tissues into a single cryoblock to enable the serial processing of many tissues (Figure 1B). We additionally aimed for simultaneous freezing of the tissue and the surrounding embedding media (OCT), removing the necessity of repeated thawing for cryosections. We designed the cryomolds using polytetrafluoroethylene (PTFE), which has ideal anti-adherence characteristics and adequate thermal conductivity (0.292W/(m*K)),[6] and its high robustness allows the molds to be re-used for >4 years without impairment. We provide a detailed protocol for MTM-assisted cryoprocessing (Figure S2; STAR Methods). In brief, the tissue is fixed, cryoprotected in 30% sucrose, transferred into OCT, and then transferred into an MTM. The blocks are partially pre-frozen, filled with OCT, and then frozen completely. The OCT block is then taken out of the mold, flipped upside down, and placed back into the MTM. The surface is then slightly heated (but not to the point of melting the block), more OCT is added, and a lid is pressed on to create a flat surface. After trimming the overhanging OCT, the blocks are ready for cryosectioning. As a proof of principle, we embedded a total of 72 cerebral organoids (2–3 mm in size, 4 per position in a 6 × 3 MTM) with either constitutive GFP (CAG-GFP), tdtomato (CAG-tdtomato), or no fluorophore, cryosectioned, and immunolabeled for GFP, tdtomato, and DAPI. Confocal spinning disk microscopy then demonstrated appropriate tissue positioning (Figure 1C). Additionally, we successfully tested the compatibility of MTMs with paraffin embedding and sectioning, as indicated by H&E staining of vibratome sectioned organoids (Figure S3). Thus, MTMs can be used to section both cryopreserved and paraffin-embedded tissues in an upscaled manner.

MTMs enable parallel heterogeneous tissue processing

To investigate if MTMs can be used to process heterogeneous tissues, we selected 19 mature mouse-derived tissue samples (femur, pancreas, skin, spleen, cerebellum, olfactory bulb, tail, testis, spinal cord, liver, stomach, eye, muscle [hindleg], colon and duodenum, kidney, heart, salivary gland, and cortex) and processed them using MTMs (Figures 2A–2D). To demonstrate the flexibility of grouping tissues, the colon and duodenum were co-embedded in the same position. Immunolabeling for E-cadherin, MAP2, CK19, and DNA (DAPI) revealed that MTM blocks can be used for the simultaneous cryoprocessing of diverse, heterogeneous tissues (Figure 2E). Notably, all tissues remained intact, and tissue-specific morphologies and marker expression were readily observable (Figure 2F). We further demonstrated the usability of MTMs in high-magnification (40×) microscopy to investigate E-cadherin and laminin expression throughout all sampled tissues. Not only does high-magnification imaging confirm the preservation of distinct structural features across each tissue, but it also underscores the consistent localization of E-cadherin and laminin within relevant anatomical niches (Figures S4A and S4B). In summary, MTMs can be used for upscaled cryoprocessing of tissues with heterogeneous features while maintaining cellular and subcellular structural details.

MTMs can be used to process heterogeneously sized tissue and investigate spatiotemporal marker expression

We next investigated if MTMs could be used to process heterogeneously sized tissue. Cerebral organoids were grown for 1–30 days, fixed, and cryoprocessed using MTMs. In total, 21 groups were analyzed (daily from day 1 to 12 and every 2 days from day 14 to 30). After processing in 3 × 7 MTMs, tissues from all time points were present in the resulting cryosections (Figures 3A–3E). We next investigated whether such tissue could be used to cost-effectively investigate the temporal expression of proteins. We performed fluorescent IHC with a range of markers associated with neural differentiation (Figures 3A–3E) and performed microscopy using an Olympus IX83 inverted

CellPress
OPEN ACCESS

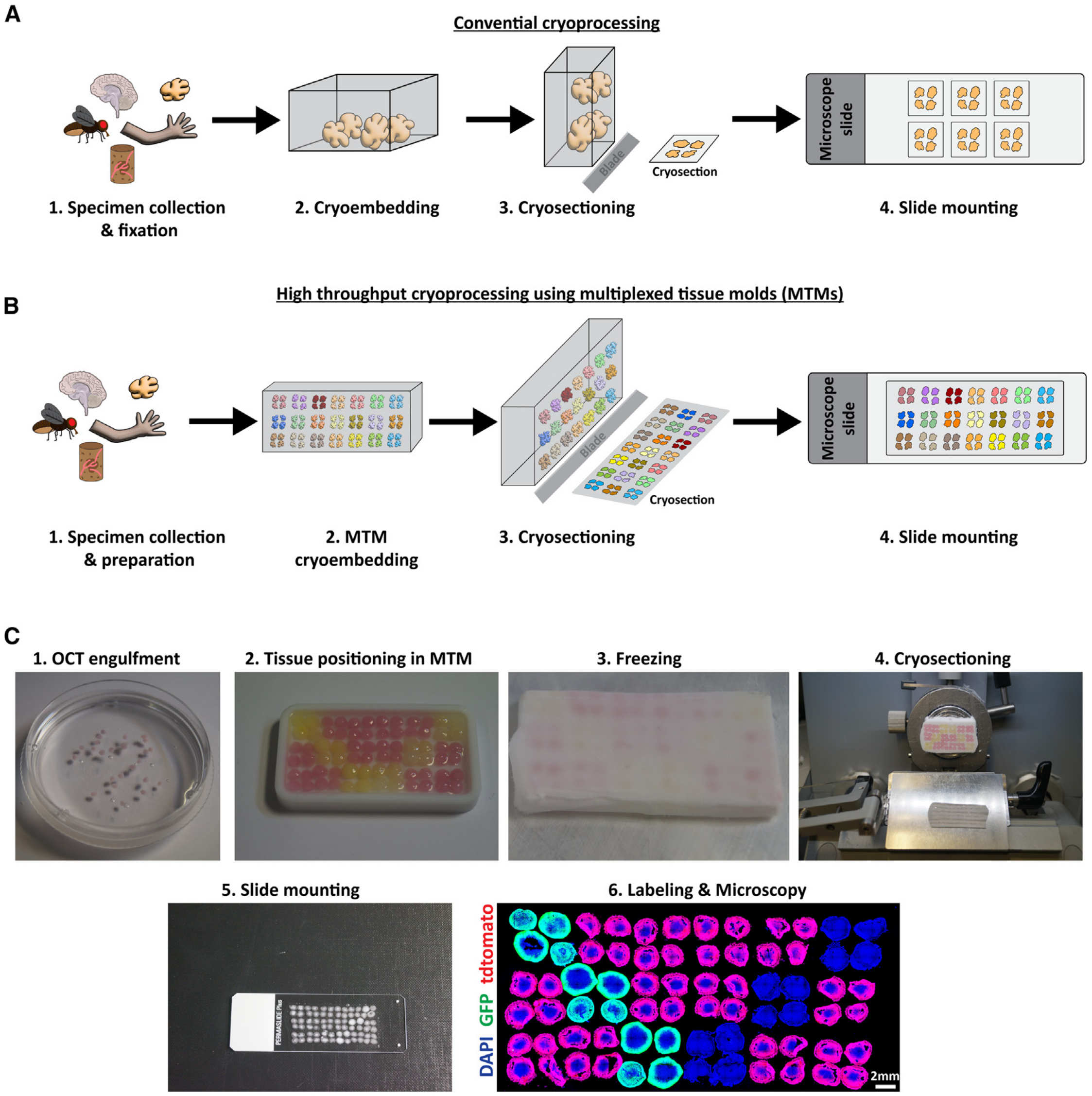

Figure 1. Multiplexed cryoprocessing of tissue

(A) Schematic of conventional tissue processing for cryoprocessing (in the example of 72 brain organoids). Specimen are collected, processed, and fixed before being positioned in a mold and embedded using an optimal cutting temperature (OCT) compound, which is then shock frozen (cryoembedded) and sectioned with a cryostat (cryosectioned). Cryosections are then directly mounted to positively charged microscopy slides.

(B) Using MTMs, the process of cryoembedding can be multiplexed at the stage of OCT embedding, which results in cryosections containing a multiplicity of tissue (up to 21× with molds used in this study).

(C) Processing of cerebral organoids with GFP, tdtomato, or no fluorophore expression. 72 cerebral organoids (~3 mm in size) were first engulfed in OCT (1) and then positioned in an MTM mold with 6 × 3 grid (4 organoids per position) (2) and frozen on a metal block on dry ice (3). Tissue was sectioned in a cryostat (4) and mounted on positively charged microscopy slides (5). Microscopy recordings for DAPI, GFP, and tdtomato reveal the correct positioning of all organoids (6).

spinning disk microscope with a low-magnification objective (10×).

We next quantified the temporal fluorescence intensity of ten markers associated with differentiation from pluripotent stem cells along the neural lineages (OCT4, E-cadherin, SOX2, N-cadherin, OTX2, SOX1, FOXG1, PAX6, MAP2, and TUBB3 [TUJ1]) throughout the time course of cerebral organoid development and corrected for DAPI intensity (Figures 3F–3O).[7–9] We readily observed a decline of the pluripotency marker OCT4 as well as the stereotypic E-cadherin-to-N-cadherin switch when differentiating pluripotent stem cells toward neural stem cells and neurons (Figures 3F–3H).[10] The pluripotency and neural stem cell marker SOX2 remained highly expressed, albeit at higher levels in neural stem cells, while SOX2 protein was absent in neurons (Figure 3I). The forebrain and midbrain neural progenitor marker OTX2 became upregulated in a similar temporal manner to N-cadherin (Figure 3J). However, at least in a forebrain context, OTX2 expression seemed to be reduced in

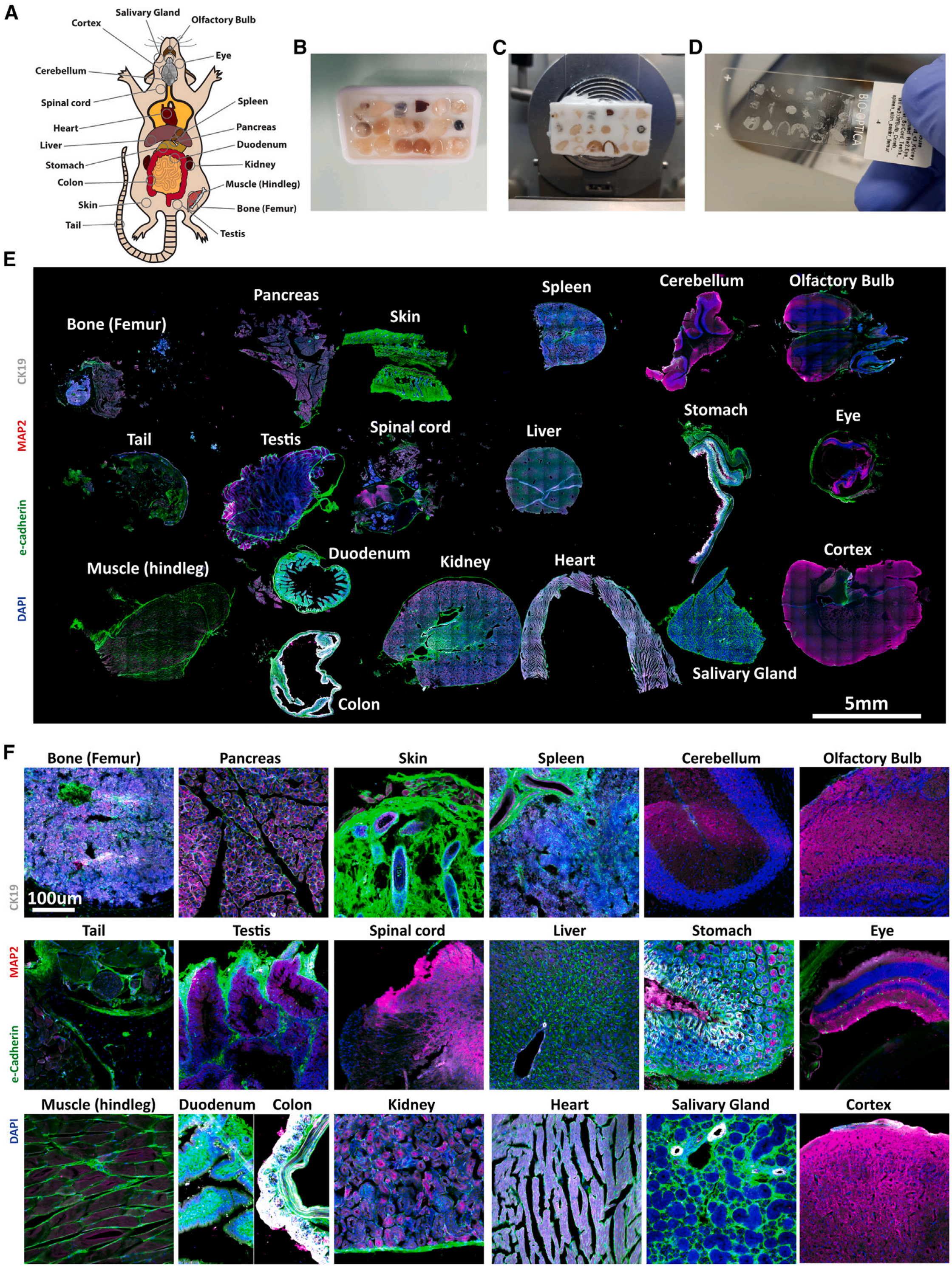

Figure 2. Efficient multiplexed cryosectioning of 19 heterogeneous tissues

(A) Selected tissues for MTM processing.

(B–D) Procedure of multiplexed tissue embedding in a 6 × 3 grid MTM. Colon and duodenum were co-embedded in one well. Femur, tail, and spinal cord were pre-processed with decalcification solution.

(E) Whole-slide stitched microscopy image of an immunostaining for E-cadherin, MAP2, CK19, and DAPI. Individual tissues are labeled within the figure.

(F) Magnified recordings of all 19 tissues displaying morphological features of individual organs. *N* = 1 mouse and 19 organs.

CellPress
OPEN ACCESS

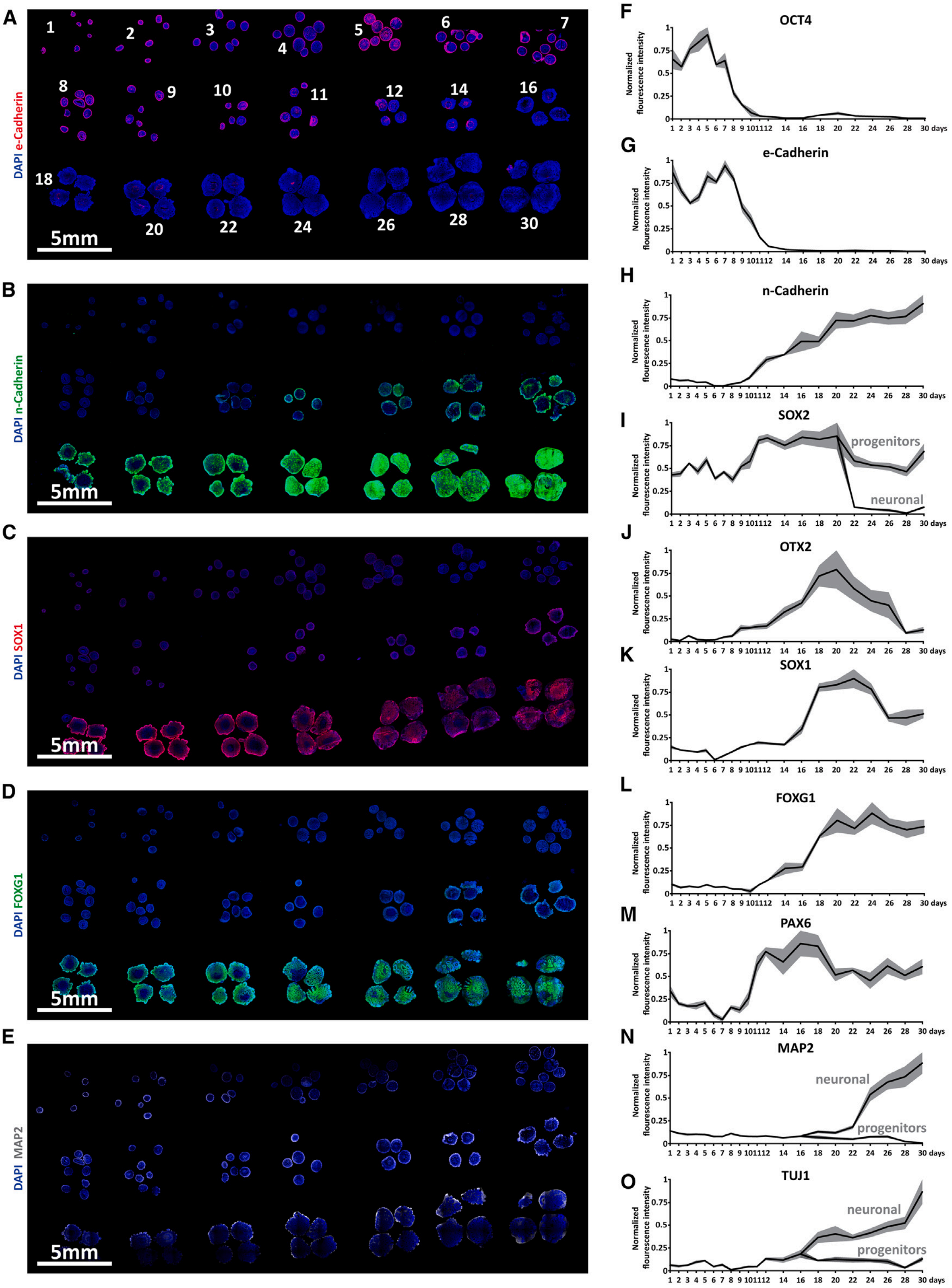

Figure 3. Time course of marker expression in cerebral organoids between days 1 and 30

(A–E) Immunolabeling for selected temporal markers of neural differentiation (E-cadherin, N-cadherin, SOX1, FOXG1, and MAP2), which were expressed in the time course of organoid differentiation between days 1 and 30. Selected time points are indicated in (A) (daily: days 1–12; every second day: days 14–30).

(legend continued on next page)

later forebrain differentiation. Interestingly, we found that SOX1 expression correlated with the expression of the forebrain marker FOXG1 and the neural stem cell (and later cortical progenitor marker) PAX6 (Figures 3K–3M). The neural markers MAP2 and TUJ1 were first upregulated in neural populations around day 16, with clear expression in neurons but not in progenitors (Figures 3N and 3O).

Notably, IHC against laminin clearly indicated the time point of the laminin-rich Matrigel addition to the media (2% Matrigel added on day 11 and laminin present in sections from day 12 onwards) (Figures 4A and 4B) but, surprisingly, also revealed the deposition of a layer of laminin around neuroepithelial rosettes, which was maintained at later stages (Figures 4B and 4C). Notably, this layer of laminin created a lower cell density boundary between the neuroepithelial tissue and the later-forming neural clusters (Figures 4D–4G) and acted as a fundamental structural component in the formation of expanded neuroepithelial rosettes, allowing rosette expansion and potentially mimicking the initial functionality of the pial basement membrane.[11] Notably, we found that this laminin coating was persistent even at later stages and defined the location of neuroepithelial rosettes, unlike the pial basement membrane in the developing brain, which is confined to the outside of the cortical plate, indicating that Matrigel addition might only model the initial functionality of the pial basement membrane in development.

In summary, MTMs allow fast and effective processing of heterogeneously sized tissue and enable the analysis of stem cell aggregate expression profiles as they develop toward neural stem cells and neurons, thus permitting cost-efficient time-course analyses of tissues on a single slide.

MTMs substantially reduce cost and labor

We next calculated the approximate cost of consumables associated with cryoprocessing in the preceding analysis (Figure 3). For consumables such as antibodies, media, and slides, aliquot costs were estimated (Table S1). While fluorescence IHC allows for multiple antibody labels per slide, this could cause significant price heterogeneity (e.g., if two antibodies are derived from the same species, then independent slides would have to be labeled). We, therefore, calculated the use of one individual antibody per slide. In total, the prices for consumables for the preceding analysis were 5.2 € (OCT), 5.1 € (microscopy slides), 1.0 € (antibody staining solution), 1.1 € (blot/perm solution), 4.1 € (PBS-T), 3.9 € (PBS), 33.5 € (primary antibodies), 2.9 € (secondary antibodies), 1.5 € (coverslips), and 2.2 € (mounting media). Thus, for the analysis of protein expression of 10 proteins across 21 time points, with 4–10 samples per time point, the total cost of consumables was only about 60.4 € (6.0 €/slide). For one individual tissue group (3 × 7 MTM), the cost of all analyses was only around 2.9 €, and for one individual organoid (of all ~114 organoids of this experiment), the cost was ~0.5 €. With antibody multiplexing (e.g., 3 antibodies/slide with 4 total slides), the price for the entire analysis of Figure 3 (10 antibodies on 4 slides) can be further reduced to about 47.5 €. In contrast, a similar analysis using conventional processing (Figure 1A) would cost between 1,349 € (one antibody per slide, 210 slides) and 1,077 € (2–3 antibodies per slide, ~84 slides) for 10 antibodies on 21 tissues/tissue groups. Thus, the usage of MTMs results in a ~96% reduction in the cost of consumables in both instances.

Furthermore, the processing of all tissues simultaneously, compared to individual processing, reduced the time spent performing cryosectioning by up to 95% (10 vs. 210 slides), with a similar increase in efficiency in tissue labeling and analysis, while being easier to handle in comparison to regular OCT blocks due to the increased size of the sections and defined tissue negative borders of the section. Scanning 4 vs. 84 slides (or 10 vs. 210 slides, if antibodies are not multiplexed) will be a dramatic time improvement in any scientific or diagnostic setup: scanning 4 slides takes ~2 h of recording time (~30 min/slide), whereas 84 slides would take 1.7 days of pure recording—excluding the slide setup in between. Similarly, when only one antibody per slide is used, the recording time differs between 5 h (MTM) vs. 4.4 days (individual tissues) of pure recording time.

An additional advantage of MTM-processed tissue is the substantial reduction in storage space required for tissue preservation. Depending on the number of sections needed from a single block, this method can achieve a comparable space reduction of up to ~95%, significantly reducing the storage-associated ecological and environmental footprint.

In summary, MTMs drastically reduce the costs of cryosectioning and speed up tissue analysis, making MTMs an ideal solution for cost-efficient and high-throughput tissue analysis.

DISCUSSION

Here, we present a streamlined, straightforward solution to high-throughput tissue processing, which permits the cost-efficient generation of multiplexed cryosections. While cryosections are the gold standard for antibody labeling or *in situ* hybridization (ISH)-based tissue analysis, as well as spatial transcriptomics, the time-consuming processing of tissue processing, as well as the expensive components, hinder this technique's wider adoption in basic research and making cryosectioning and IHC a routine application in diagnostics. We show that multiplexing with MTMs dramatically reduces the time and cost of cryosectioning, which will help laboratories in both research and diagnostic environments drastically reduce costs. The general workflow of MTMs is compatible with tissue processing for ISH-based methodologies, which would enable high-throughput transcriptomic readouts. MTMs could also easily be adapted to be used for, e.g., precise positioning of tissue sections for spatial transcriptomics approaches.

Furthermore, the stereotypic positioning of tissues in MTM-derived sections would permit the development of automated

(F–O) Quantification of fluorescence intensity of selected markers in the time course of cerebral organoid differentiation. For SOX2, MAP2, and TUJ1, fluorescence intensity was quantified for both neural progenitors and neurons after fluorescence intensity became distinguishable. Fluorescence intensity was normalized first to DAPI intensity and then normalized to the highest value in the time course. Gray bars display SEM. *n* = 3–9 organoids of one experiment per time point with sections on the same slide.

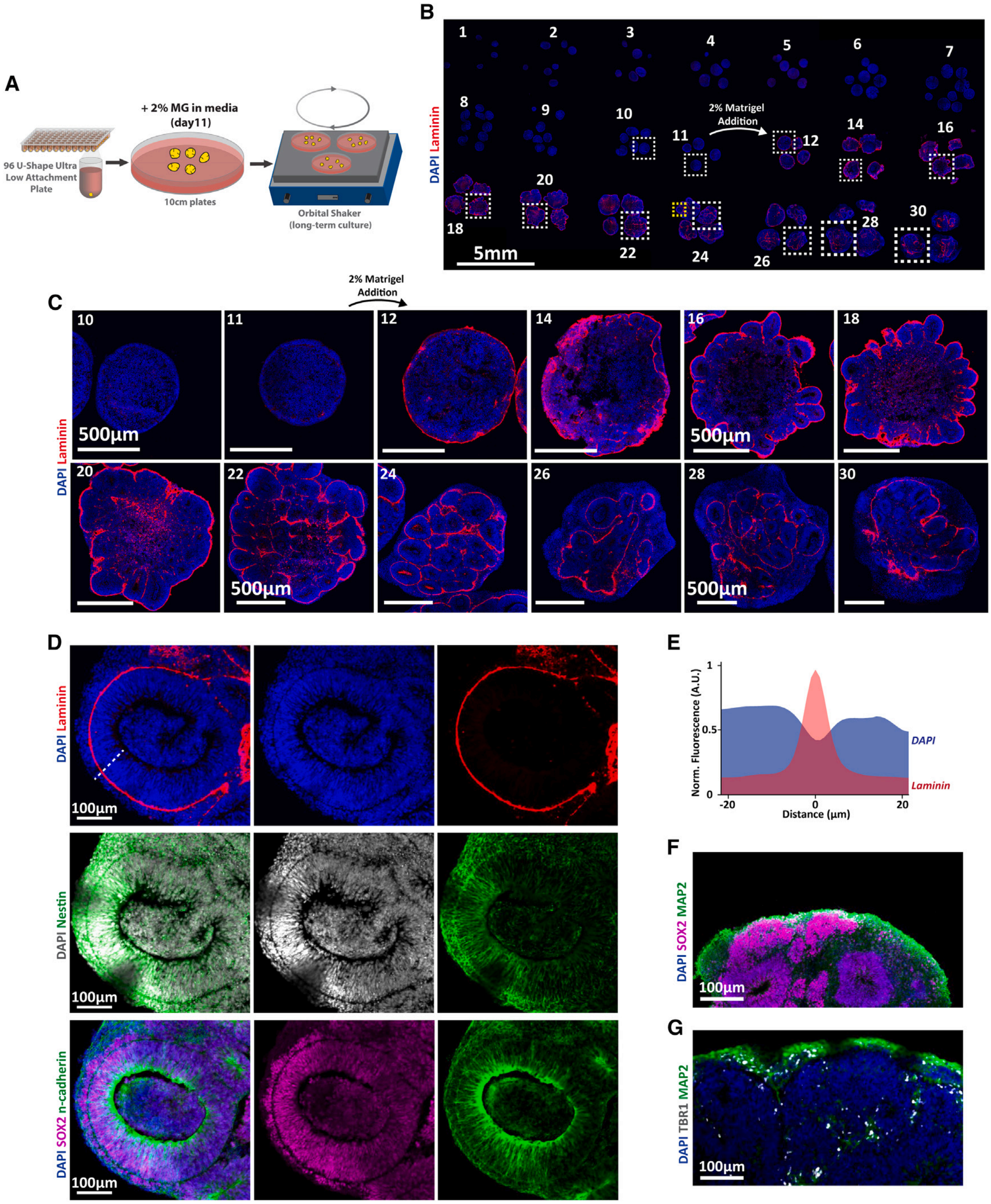

Figure 4. 2% Matrigel addition results in maintained ECM deposition around neural rosettes

(A) Schematic of the organoid protocol and addition of Matrigel.

(B) Whole-slide scan of cerebral organoid time course with fluorescent labeling of laminin and DNA (DAPI).

(C) Magnified images of (B) (dashed boxes).

(D) Magnified view of a neural rosette with laminin (top), Nestin (middle), and SOX2 and N-cadherin (bottom) labeling from serial sections on day 24 (yellow dashed line in B).

(E) Normalized fluorescence intensity profile over distance of both DAPI as well as laminin (n = 44 representative lines of interest of 4 organoids on day 24, representative line: white dashed line in D).

(F) Representative image of a 24-day-old organoid with fluorescent immunolabeling for SOX2 and MAP2.

(G) Representative image of a 24-day-old cerebral organoid with immunolabeling for TBR1 and MAP2. n = 4–9 organoids of one experiment per time point with sections on the same slide.

pipelines to detect tissues in specific positions and process/analyze them separately. Developing automated tissue processing based on MTMs, as well as AI-assisted algorithms for image analysis and interpretation, could further reduce the cost associated with tissue analysis, particularly in diagnostics laboratories.

Currently, multiplexing is limited to tissues but not antibody labeling readouts. Thus, further developments of this technique could include the addition of additional sample loading mechanisms as well as the introduction of a platform that enables immunolabeling of each individual grid position separately, allowing different sets of antibodies to be used on different tissues on the same slide. This would allow, for example, a group of heterogeneous patient biopsies to be independently processed for tissue- and patient-specific disease markers. Parallel processing of multiple tissues, such as in the example of 19 mouse organs, will also provide a robust and cost-efficient system for antibody validation experiments, for simultaneous study of heterogeneous *in vitro* tissues, and for investigation of the effects of genetic, chemical, or other perturbations across multiple tissues. Further adaptations to the shape and size of MTMs could be used to process even more (or larger) tissues, which could decrease cost and labor even further. Additionally, MTMs could be designed in various shapes, which could hold tissues in space stereotypically, where all tissues are precisely positioned, to cut precisely through the same positions of all tissues simultaneously. Finally, MTMs are made out of extremely durable and deformation-resistant PTFE, which will drastically reduce plastic waste (and costs) in comparison to single-use plastic embedding molds.

Limitations of the study

Current limitations of the method are the more labor-intensive positioning of tissues into MTMs, which can—if not performed carefully—result in the mispositioning of the tissue, which is irreversible. For particularly transparent or white tissues or those that are hard to see, MTMs could be manufactured in other colors (e.g., black), which could help to increase the contrast to the tissue, as the current white material results in the limited visibility of particularly small tissues. While only one animal has been used in this study, we do not believe that this will affect the robustness of the presented methodology. While we have tested MTM-based cryosectioning using gelatin embedding (data not shown), we recommend OCT due to the stability and flexibility concerns of larger gelatin slabs, which might damage the tissues and were generally harder to handle.

RESOURCE AVAILABILITY

Lead contact

Requests for resources of this article and any additional information should be directed to the lead contact, Jürgen A. Knoblich (juergen.knoblich@imba.oeaw.ac.at).

Materials availability

The authors are working on the commercialization of MTMs to make the product available to the scientific community. In the meantime, we provide all the information necessary for other scientists to generate MTMs, provided equipment availability. The necessary CAD files for MTMs are provided in Data S1. No other materials have been generated in this study.

Data and code availability

- Microscopy data used in this paper will be shared upon request by the lead contact.
- This paper does not report original code.
- Any additional information required to reanalyze the data reported in this work is available from the lead contact upon request.

ACKNOWLEDGMENTS

The authors thank all current and past members of the Knoblich laboratory and Vienna BioCenter (VBC) for technical expertise and manuscript feedback, especially Peter-Christopher Esk, Jaydeep Sidhaye, Sakuraku Wong, Chong Li, Francois Bonnay, and Christian Krauditsch. The authors thank the Mechanical Engineering Center, particularly Martin Colombini, for design and material discussions and manufacturing of MTMs. The authors also thank the IMBA/IMP BioOptics facility for microscopy services and discussions, particularly P. Pasierbek and A. Moreno Cencerrado; the IMBA IPSC Biobank and IMBA Stem Cell Core Facility (https://www.oeaw.ac.at/imba/scientific-facilities/stem-cell-core-facility) for generation of iPSC lines; the Ethics and Biosafety Department for coordination of ethics approvals; and the VBC Core Facilities (VBCF), particularly the Histology Facility, for discussions and sectioning support (https://www.viennabiocenter.org/). Work in the laboratory of J.A.K. was supported by the Austrian Federal Ministry of Education, Science and Research; the Austrian Academy of Sciences; the City of Vienna; a European Research Council (ERC) Advanced Grant (no. 695642) under the European Union's Horizon 2020 Programme; the Austrian Science Fund (FWF) (Special Research Program F7804-B and Stand Alone grants P 35680 and P 35369); and funding by the Austrian Lotteries. Work by D.R. was supported by the ERC Advanced Grant (no. 695642), the FWF P 35369 grant, and the Austrian Lotteries.

AUTHOR CONTRIBUTIONS

D.R. and J.A.K. conceived and planned the project and wrote the manuscript with support from all the authors. D.R. performed tissue culture, tissue processing, labeling, and quantifications. M.C. consulted on MTM design and instructed the manufacturing of MTMs in the VBC Mechanical Engineering Center. P.M. performed the animal work. A.P. assisted with vibratome sectioning of paraffin blocks. Funding was acquired by J.A.K. and D.R.

DECLARATION OF INTERESTS

J.A.K. is an inventor on a patent describing cerebral organoid technology (European patent application no. EP2743345A1) and a cofounder and member of the scientific advisory board of a:head bio AG. J.A.K. and D.R. are inventors on a patent application describing brain organoid fusion technology (patent application no. EP22177191.8) and a patent describing organoid technology (patent submission ID: GB2206768.0). J.A.K. and D.R. are inventors on a patent application describing MTM technology (European patent application no. EP 24191197.3).

STAR★METHODS

Detailed methods are provided in the online version of this paper and include the following:

- KEY RESOURCES TABLE
- EXPERIMENTAL MODEL AND STUDY PARTICIPANT DETAILS
  - Stem cell culture details
  - Cerebral organoid generation
  - Organoid media
  - Mouse tissue preparation
- METHOD DETAILS
  - Tissue pre-processing
  - MTM manufacturing
  - MTM tissue embedding
  - Cryosectioning & slide storage

- Immunohistochemistry
- Microscopy
- Image processing
- QUANTIFICATION AND STATISTICAL ANALYSIS
  - Fluorescence quantification

SUPPLEMENTAL INFORMATION

Supplemental information can be found online at https://doi.org/10.1016/j.crmeth.2025.101023.

Received: October 1, 2024
Revised: February 1, 2025
Accepted: March 20, 2025
Published: April 21, 2025

REFERENCES

1. Kim, S.W., Roh, J., and Park, C.S. (2016). Immunohistochemistry for Pathologists: Protocols, Pitfalls, and Tips. J. Pathol. Transl. Med. *50*, 411–418.
2. Buchwalow, I.B., and Böcker, W. (2010). Immunohistochemistry: Basics and Methods (Springer Science & Business Media).
3. Kim, J., Koo, B.K., and Knoblich, J.A. (2020). Human organoids: model systems for human biology and medicine. Nat. Rev. Mol. Cell Biol. *21*, 571–584.
4. Masselink, W., Reumann, D., Murawala, P., Pasierbek, P., Taniguchi, Y., Bonnay, F., Meixner, K., Knoblich, J.A., and Tanaka, E.M. (2019). Broad applicability of a streamlined ethyl cinnamate-based clearing procedure. Development *146*, dev166884.
5. Kononen, J., Bubendorf, L., Kallioniemi, A., Bärlund, M., Schraml, P., Leighton, S., Torhorst, J., Mihatsch, M.J., Sauter, G., and Kallioniemi, O.P. (1998). Tissue microarrays for high-throughput molecular profiling of tumor specimens. Nat. Med. *4*, 844–847.
6. Overview of materials for Polytetrafluoroethylene (PTFE), Extruded. https://www.matweb.com/search/datasheet_print.aspx?matguid=4e0b2e88eeba4aaeb18e8820f1444cdb
7. Renner, M., Lancaster, M.A., Bian, S., Choi, H., Ku, T., Peer, A., Chung, K., and Knoblich, J.A. (2017). Self-organized developmental patterning and differentiation in cerebral organoids. EMBO J. *36*, 1316–1329.
8. Eze, U.C., Bhaduri, A., Haeussler, M., Nowakowski, T.J., and Kriegstein, A.R. (2021). Single-cell atlas of early human brain development highlights heterogeneity of human neuroepithelial cells and early radial glia. Nat. Neurosci. *24*, 584–594.
9. Pan, G.J., Chang, Z.Y., Schöler, H.R., and Pei, D. (2002). Stem cell pluripotency and transcription factor Oct4. Cell Res. *12*, 321–329.
10. Miyamoto, Y., Sakane, F., and Hashimoto, K. (2015). N-cadherin-based adherens junction regulates the maintenance, proliferation, and differentiation of neural progenitor cells during development. Cell Adhes. Migrat. *9*, 183–192.
11. Halfter, W., Dong, S., Yip, Y.P., Willem, M., and Mayer, U. (2022). A Critical Function of the Pial Basement Membrane in Cortical Histogenesis. J. Neurosci. *22*, 6029–6040.
12. Reumann, D., Krauditsch, C., Novatchkova, M., Sozzi, E., Wong, S.N., Zabolocki, M., Priouret, M., Doleschall, B., Ritzau-Reid, K.I., Piber, M., et al. (2023). In vitro modeling of the human dopaminergic system using spatially arranged ventral midbrain–striatum–cortex assembloids. Nat. Methods *20*, 2034–2047.

STAR★METHODS

KEY RESOURCES TABLE

REAGENT or RESOURCE	SOURCE	IDENTIFIER
Antibodies		
Mouse anti *E*-Cadherin (1:100)	BD Biosciences	Cat.# 610182; RRID:AB_397581
Rabbit anti CK19 (1:500)	Abcam	Cat.# AB52625; RRID:AB_2281020
Rabbit anti OCT-4 (1:100)	Cell Signaling Technologies	Cat.# 2840; RRID:AB_2167691
Goat anti SOX2 (1:100)	R&D Systems	Cat.# AF2018; RRID:AB_355110
Goat anti n-Cadherin (1:500)	BD Biosciences	Cat.# 610920; RRID:AB_2077527
Goat anti OTX2 (1:100)	R&D Systems	Cat.# AF1979; RRID:AB_2157172
Goat anti SOX1 (1:500)	R&D Systems	Cat.# AF3369; RRID:AB_2239879
Rabbit anti FOXG1 (1:200)	Abcam	Cat.# Ab18259; RRID:AB_732415
Sheep anti PAX6 (1:200 of 100ul reconstitute)	R&D Systems	Cat.# AF8150; RRID:AB_2827378
Chicken anti MAP2 (1:500)	Abcam	Cat.# Ab5392; RRID:AB_2138153
Mouse anti TUJ1 (βIII-tubulin) (1:500)	Sigma-Aldrich	Cat.# T8578-100UL; RRID:AB_1841228
Rabbit anti Laminin (1:100)	Abcam	Cat.# AB11575; RRID:AB_298179
Donkey anti mouse AF488 (1:500)	Invitrogen	Cat.# A21202; RRID:AB_141607
Donkey anti mouse AF568 (1:500)	Invitrogen	Cat.# A10037; RRID:AB_11180865
Donkey anti mouse AF647 (1:500)	Invitrogen	Cat.# A31571; RRID:AB_162542
Donkey anti rabbit AF488 (1:500)	Invitrogen	Cat.# A21206; RRID:AB_2535792
Donkey anti rabbit AF568 (1:500)	Invitrogen	Cat.# A10042; RRID:AB_2534017
Donkey anti rabbit AF647 (1:500)	Invitrogen	Cat.# A31573; RRID:AB_2536183
Donkey anti chicken AF488 (1:500)	Jackson ImmunoResearch	Cat.# 703-545-155; RRID:AB_2340375
Donkey anti chicken AF647 (1:500)	Jackson ImmunoResearch	Cat.# 703-605-155; RRID:AB_2340379
Donkey anti Sheep AF488 (1:500)	Invitrogen	Cat.# A11015; RRID:AB_2534082
Donkey anti Sheep AF568 (1:500)	Invitrogen	Cat.# A21099; RRID:AB_10055702
Donkey anti Sheep AF647 (1:500)	Jackson ImmunoResearch	Cat.# 713-605-147; RRID:AB_2340751
Donkey anti Goat AF488 (1:500)	Invitrogen	Cat.# A11055; RRID:AB_2534102
Donkey anti Goat AF568 (1:500)	Invitrogen	Cat.# A11057; RRID:AB_2534104
Donkey anti Goat AF647 (1:500)	Invitrogen	Ca.t# A21447; RRID:AB_2535864
Chemicals, peptides, and recombinant proteins		
EDTA	Sigma-Aldrich	Cat.# E6758
mTESR1	Stemcell Technologies	Cat.# 85875
DPBS−/−	ThermoFisher	Cat.# 14190250
Anti-adherence rinsing solution	Stemcell Technologies	Cat.# 07010
DMEM/F12	Invitrogen	Cat.# 11330-057
N2 supplement	ThermoFisher	Cat.# 17502001
GlutaMax-I	ThermoFisher	Cat.# 35050-038
MEM-NEAA	Sigma-Aldrich	Cat.# M7145
Neurobasal	Gibco	Cat.# 21103049
B27-A	ThermoFisher	Cat.# 12587010
Insulin	Sigma-Aldrich	Cat.# I9278
OCT	Fisher Scientific	Cat.# 12351753
Accutase	Sigma-Aldrich	Cat.# A6964
Y27632 Rock Inhibitor	Selleck Chemicals	Cat.# S1049
Heparin solution	Sigma-Aldrich	Cat.# H3149-100KU
PenStrep	Sigma-Aldrich	Cat.# P4333
Antibiotic-Antimycotic	Thermo Fisher	Cat.# 15240062

(*Continued on next page*)

CellPress
OPEN ACCESS

Continued

REAGENT or RESOURCE	SOURCE	IDENTIFIER
hES-qualified Matrigel	Corning	Cat.# 354277
Matrigel	Corning	Cat.# 356235
Vitamin C	Sigma-Aldrich	Cat.# A4544
Sodium Bicarbonate	Sigma-Aldrich	Cat.# S5761
16% Formaldehyde solution	Sigma-Aldrich	Cat# 28906
BSA	Thermo Fisher	Cat.# 11021037
TX100	Sigma-Aldrich	Cat.# 93420
DAKO Mounting Media	Agilent	Cat.# S3023
Experimental models: Cell lines		
WA09 (H9) WT	WiCell	WA09; RRID:CVCL_9773
WA09 (H9) CAG-GFP	This paper	N/A
WA09 (H9) CAG-tdtomato	This paper	N/A
Experimental models: Organisms/strains		
C57BL/6J (1 male mouse)	IMP Transgenic Core Facility	C57BL/6J (e.g., Jackson Laboratory: Cat.# 000664)
Software and algorithms		
Fiji (v1.53q)	https://imagej.net/software/fiji/	V1.53q
Other		
PTFE (Senoflon) extruded PTFE sheets	Senova	N/A

EXPERIMENTAL MODEL AND STUDY PARTICIPANT DETAILS

Stem cell culture details

The hESC line H9 (WA09, WiCell) as well as CAG-GFP and CAG-tdtomato (in WA09 genomic background) were cultured feeder-free on hESC-qualified Matrigel (Corning, Cat.#354277) in mTESR1 (Stremcell Technologies, Cat.#85875) in a cell culture incubator (humidified, 37°C, 5% CO_2). Cells were routinely tested for genome integrity and Mycoplasma contamination. Cells were split when colonies were not yet showing a dense colony core and before colony fusion, usually after 3–5 days. Splitting was performed by incubation in an incubator (humidified, 37°C, 5% CO_2) in DPBS$^{-/-}$ containing 0.5mM EDTA (DPBS: Thermo Fisher, Cat.# 14190250, EDTA: Sigma-Aldrich, Cat.#E6758) until cracks in the center of the colonies appeared (usually after 4–8 min). PBS was then aspirated and colonies gently washed off with 1mL mTESR1. Stem cell tissue clumps were then transferred in a ratio of 1:6 to 1:10 into Matrigel coated plates. For Matrigel coating, 1mL mTESR1 containing Matrigel (see producer instructions for batch-dependent amount of Matrigel to add) was added to plates which were then incubated at 37°C for at least 30min or alternatively sealed with Parafilm and stored in a fridge for up to 2 weeks. Before usage, fridge-cold plates were warmed in an incubator for at least 15 min.

Cerebral organoid generation

Organoids have been generated as reported before.[12] An overview of used media can be found below ("Organoid media" section). In brief, human embryonic stem cells were used 2–3 days after splitting. Cells were rinsed once with PBS and then incubated with 600 μL Accutase (Sigma-Aldrich, Cat.# A6964) (volume for a well of a 6-well plate) until all cells detach after slightly tapping the side of the plate. Cells are then gently pipetted up and down using a P1000 pipet tip and transferred into a 15mL falcon tube with 9mL mTESR1. Cells are then centrifuged at 200g for 5min and the cell pellet is resuspended in 1mL mTESR1 containing 1:100 ROCK (Rho kinase) inhibitor (RI) Y27632 (Selleck Chemicals, Cat.#S1049) and counted using a Countess 3 cell counter. Cells were only used if the live count was >90%. 9000 hESCs were transferred into 150μL stem cell media (1:100 RI and optional PenStrep) per well (e.g., for a 96 well plate, 100 wells were prepared, which corresponds to 15mL mTESR1 and 900.000 cells). Media was gently mixed and pipetted into ultra-low attachment plates (Thermo Fisher Scientific, Cat.# 136101 and Corning Cat.# 7007) and incubated in a tissue culture incubator for 3 days 100μL of media was exchanged on day 3 with 150μL of fresh mTESR1 (without RI). On day 5, 7, 9 (optional: day 11), 150μL of media were exchanged with Neural Induction (NI) media. On day 11–13, up to 30 embryoid bodies (EBs) were transferred into 10cm plates which were coated prior with anti-adherence rinsing solution (Stemcell Technologies, Cat.# 07010). 10mL Improved-A media containing 2% Matrigel (Corning, Cat.# 356235, added to cold media and used within 1h) was then added to the EBs. For further differentiation of brain organoids, media was exchanged after 3 days with Improved -A media

without Matrigel, with changing to Improved +A media around day 16. Organoids were transferred to orbital shakers (Inforce Celltron HT) with reduced shaking speed (42 rpm) on day 20. From the timepoint organoids were transferred on the shaker, 30mL of media was added per 10cm plate.

Organoid media

Neural induction (NI) media: DMEM/F12 (Invitrogen, Cat.# 11330-057), 1% N2 Supplement (ThermoFisher, Cat.# 17502001), 1% GlutaMAX-I (ThermoFisher, Cat.# 35050-038), 1% MEM-NEAA (Sigma-Aldrich, M7145), 1:1000 Heparin solution (Sigma-Aldrich, Cat.#H3149-100KU), 1% PenStrep (Sigma-Aldrich, Cat.#P4333)

Improved-A media: 50:50 DMEM/F12:Neurobasal (Gibco, Cat.# 21103049). 0.5% N2 supplement, 2% B27-A (ThermoFisher, Cat.# 12587010), 1:4000 Insulin (Sigma-Aldrich, I9278), 1% GlutaMAX, 0.5% MEM-NEAA, 1% Antibiotic-Antimycotic (ThermoFisher, Cat.#15240062)

Improved +A media: 50:50 DMEM/F12:Neurobasal (Gibco, Cat.# 21103049). 0.5% N2 Supplement, 2% B27 + A (ThermoFisher, Cat.# 17504044), 1:4000 Insulin (Sigma-Aldrich, I9278), 1% GlutaMAX, 0.5% MEM-NEAA, 1% Antibiotic-Antimycotic (ThermoFisher, Cat.#15240062), 1% Vitamin C solution (40mM stock in DMEM/F12) (Vitamin C: Sigma-Aldrich, Cat.# A4544), 1 g/L sodium bicarbonate (Sigma-Aldrich, Cat.#S5761)

Mouse tissue preparation

Animal experiments were approved by the Austrian Animal Care and Use Committee (approval number BMBWF-66.015/0040-V/3b/2019, TV-27). One individual mouse (C57BL/6J, male) was first euthanized with Ketamin/Xylazin. Transcardial perfusion was performed with warm PBS followed by ice-cold 4% PFA. Tissue biopsies of selected organs were then taken and processed as described below. A single mouse has been considered sufficient for this experimental setup and no reproducibility issues are expected.

METHOD DETAILS

Tissue pre-processing

Tissue was fixed in 4% formaldehyde solution (mixed from 16% stock, ThermoScientific Cat# 28906) in PBS at room temperature for 4h on a tube rotator or at 4°C over night. Optionally, tissue containing bone was extensively rinsed in PBS and then transferred into decalcification solution (Sigma-Aldrich, D0818) for 1h (note: decalcification times are tissue dependent) and then washed in PBS. Attention: Mixing of formaldehyde and decalcification solution should be avoided as formaldehyde reacts with components in the decalcification solution and forms bis-chloromethyl ether, a potent carcinogen!

After fixation (and optional decalcification), tissue was either rinsed with PBS and stored at 4°C, or directly transferred into 30% sucrose in PBS for cryoprotection and transferred onto a tube rotator or a Tilt/roller mixer at 4°C over night.

MTM manufacturing

CAD design of MTMs (see Data S1) were generated in Solid Edge and precision engineered using extruded PTFE sheets (Senoflon by Senova) and CNC machined using a 3-axis precision milling machine in vertical design (PICOMAX 56L TOP). The PTFE block was clamped in the machine vice and the contour of the part was milled out. As a final step, the part was separated from the rest of the block using a special milling cutter. Manufacturing fulfilled DIN ISO 2768-mH and DIN EN ISO 14405. The dimensions of MTMs were 46 × 28mm (wall thickness 2mm) with a total height of 6.5mm (bottom thickness 2mm). Well depth was 0.6mm, radius of wells was: 2 × 4: Well diameter of 8mm (1mm gap between wells), 3 × 6: well diameter of 5mm (1mm gap between wells), 7 × 3: well diameter of 5mm (0 or 0.4mm gap between wells). Lid dimensions were 52 × 34 × 5mm with R7 radius for corners. MTM edge corners were rounded with R1.5.

MTM tissue embedding

Tissue was processed as depicted in Figure S2. First, a box of dry ice is prepared with a flat, horizontally positioned metal block (custom design, similar to the backside of a Fisher Scientific MultiBlock 88870113) inside, which is allowed to cool down prior to usage. Notably, a metal block of >1kg weight and flat continuous surface is recommended to guarantee fast cooling and efficient processing of MTMs. Tissue was first transferred into wells or plates which were pre-filled with 3-5mL OCT (Sakura Finetek Tissue-Tek O.C.T. Compound, Fisher Scientific Cat.# 12351753). The tissue was then gently moved around in OCT using a P200 or P1000 pipet tip until water swirls were no longer visible. This step was repeated again after 2 min, to guarantee adequate engulfment of tissue with OCT. Tissue was then transferred into MTM molds with either a P200 or P1000 (dependent on tissue size) as seen in step 2 of Figure S2, while aiming to reduce the amount of transferred OCT as much as possible. Dependent on size of the specimen and experimental setup, either individual specimen or whole specimen groups were transferred together into one position (Step 3 of Figure S2). Notably, the transfer process should be performed quickly, as OCT will dry out over time. Samples should be positioned in a pattern into the MTM so that they can NOT be mirrored along the horizontal or vertical axis, to prevent miss-identification of tissues after sectioning (unless a tissue identifier, such as a fluorophore or different tissue morphologies are present). Notably, for very small structures (~500μm class) it is particularly crucial to transfer only very small

volumes of OCT, as an overfilling of OCT might result in leaking of OCT into other wells, which can carry small tissues with it and might mix up sample groups. After tissue transfer, the MTM with tissue was positioned on the metal block on dry ice until the OCT in all (!) wells starts to freeze on the outside (Step 4 of Figure S2). It is crucial with this step to balance the freezing: ideally, a ring of frozen OCT appears on the outside of each well or at the bottom, and the tissue should not be completely frozen as this might cause not adequate combination with the rest of the OCT block in the next step. When a ring of OCT appears on all tissues, the MTM is next slightly overfilled (!) with OCT (Step 5 of Figure S2) and the lid is pressed on (Step 6 of Figure S2). The MTM was then surrounded with dry ice for faster freezing. After 5-7min, the lid should easily come off the MTM (Step 7–8 of Figure S2). If the lid does not separate, the block should be kept further on dry ice for another minute. The OCT overhang can be broken off and the OCT block containing the specimen can be taken out of the MTM by slightly bending the MTM on one side, until the OCT block detaches (Step 9–10 of Figure S2). After removal of potentially overhanging OCT, the block is then flipped around and positioned back into the MTMs (Step 11 of Figure S2). The surface is then slightly melted using either a heat gun (TACKLIFE HGP70AC 230V) or touching (gently!) with the palm of a hand. Note that in this step, the aim is not to melt the block at all, but only create a very fine layer of slightly molten OCT on the outside of the block. Melting too much OCT can result in surface damage of the tissue, which should be prevented (but can be trimmed during sectioning, should it happen). More OCT is then added on top of the block and the lid is carefully pressed on (Step 12 of Figure S2). The block is then allowed to freeze fully again (3–4 min, optionally the block can be surrounded with dry ice) and the lid is removed again. After breaking off large overhang OCT and removal out of the MTM and trimming off the OCT overhang with a scissor (Step 14–15 of Figure S2), the OCT block can be either processed directly in a cryostat or wrapped in aluminum foil and stored below -70°C for several months. (Note: OCT is corrosive to metal, so scissors should be rinsed with water after trimming). Paraffin embedding was performed using the same steps, but with an increase in heat to liquefy paraffin.

Cryosectioning & slide storage

Cryosectioning was performed using an Epredia CryoStar NX70 cryostat microtome (Fisher Scientific). Blocks were mounted on sample holders using OCT. Prior to cutting, blocks were precisely aligned along the x and y axis to guarantee that all tissues are processed simultaneously (Step 16 of Figure S2). Cryosections were performed at a chamber temperature of approximately -13 to -10°C (dependent on cryostat, humidity, chamber temperature, controllable features such as blade and specimen temperature, this temperature might change significantly and must be optimized) and a specimen temperature of $\sim 2^{\circ}$C below (thus, -15 to -12°C) (Step 17 of Figure S2). Notably, this temperature is highly dependent on tissue features and for example high-lipid tissues might require much lower temperatures for processing. Sections were routinely cut at 20μm section thickness. Cryosections were flipped with a tweezer or brush (see Step 17 of Figure S2) and transferred onto positively charged microscopy slides by briefly dropping the room temperature microscopy slide onto the cryosection (a final microscopy slide can be seen in Step 18 of Figure S2). Microscopy slides were then dried in a slide box overnight and stored long-term at -20°C.

Immunohistochemistry

For antibody labeling, slides were thawed and dried at room temperature for at least 1h. Slides were then transferred into a slide rack in PBS and transferred onto an orbital shaker with slow rotation speed for 5 min to gently wash off OCT. Permeabilization and blotting was performed using perm/blot solution (PBS with 5% BSA (Thermo Fisher, Cat.# 11021037) and 0.3% TX100 (Sigma-Aldrich, Cat.# 93420), sterile filtered and frozen until use) which is directly pipetted onto the slides in a humidified staining chamber and incubated for 30min-2h. Primary antibodies were prepared in antibody staining solution (PBS with 5% BSA and 0.1% TX100, sterile filtered and frozen until use) at desired dilution (see key resources table for list of antibodies and dilution). Primary antibody solution was added after removal of perm/blot solution in a cold-room (4°C) and 200-300μL of antibody solution was used per slide. After incubation overnight in a humidified chamber, the slides were transferred into a slide rack and rinsed 3x with PBS. Then, the slides were washed 3x in PBS-T (PBS and 0.01% TX100) for 10-15min per wash. Secondary antibodies (see key resources table for list of antibodies) were prepared in a 1:500 dilution in staining solution and slides were incubated with secondary antibodies for 2h at room temperature in a humidified chamber. The antibody solution was then removed from the slide and DAPI (2 μg/mL in PBS) was added onto the tissue for 7 min at room temperature in a humidified chamber. Slides were then rinsed 3x in PBS and washed 2x in PBS-T and one last time in PBS without TX100. Slides were then mounted using DAKO mounting media (Agilent, Cat.#S3023) and dried at room temperature for at least 4h. Slides were stored at 4°C until performing microscopy.

Microscopy

Microscopy was performed using an Olympus Spinning Disk system based on the Olympus IX3 Series (IX83) inverted microscope. The system is equipped with a Yokogawa W1 spinning disk unit using 405nm, 488nm, 561nm and 640nm lasers and a dual Hamamatsu Orca Flash 4.0 camera for recording. The objectives used were 10x/NA0.3 (air) with a working distance (WD) of 3.1mm and 20x/NA0.75 (air) WD 0.6mm. For imaging of large areas, the "Focus Map" feature (Low-Density Focus map) of cellSens was used to maintain focus throughout the slides. Where required, z-stacks were recorded and maximum intensity projections were performed. A 4-channel recording ($\sim$28.000 × 11.500 pixels) resulted in a tiff file of about 2.4GB.

Image processing

Images were processed using the FIJI distribution of the open-source image processing application ImageJ (v1.53q).

QUANTIFICATION AND STATISTICAL ANALYSIS

Fluorescence quantification

Fluorescence quantification was performed by selecting the entire organoid as region of interest (ROI) of the entire organoid, or of sections of interest (e.g., avoiding the necrotic core which was identified by increased DAPI intensity and fragmented nuclear morphology and lack of positive fluorescence for any used markers), or focusing on SOX2 positive for neural progenitor and MAP2/TUJ1 positive regions for neuronal ROIs). Signal intensity was first normalized to DAPI signal per ROI and then normalized to the highest value in the time course (thus, signal intensity is displayed in a range from 0 to 1). For plotting of intensity profiles over distance (Figure 4), representative line intensity profiles were measured for each channel using the ‘‘Plot Profile’’ function of Fiji. The dataset was then processed in python (v3.9, using pandas, numpy, matplotlib and scipy.ndimage) and a Gaussian smoothing (sigma = 2) was applied before normalization and averaging.

Cell Reports Methods, Volume *5*

Supplemental information

A cost- and time-efficient method

for high-throughput cryoprocessing

and tissue analysis using multiplexed tissue molds

Daniel Reumann, Martin Colombini, Paul Möseneder, Agnieszka Piszczek, and Jürgen A. Knoblich

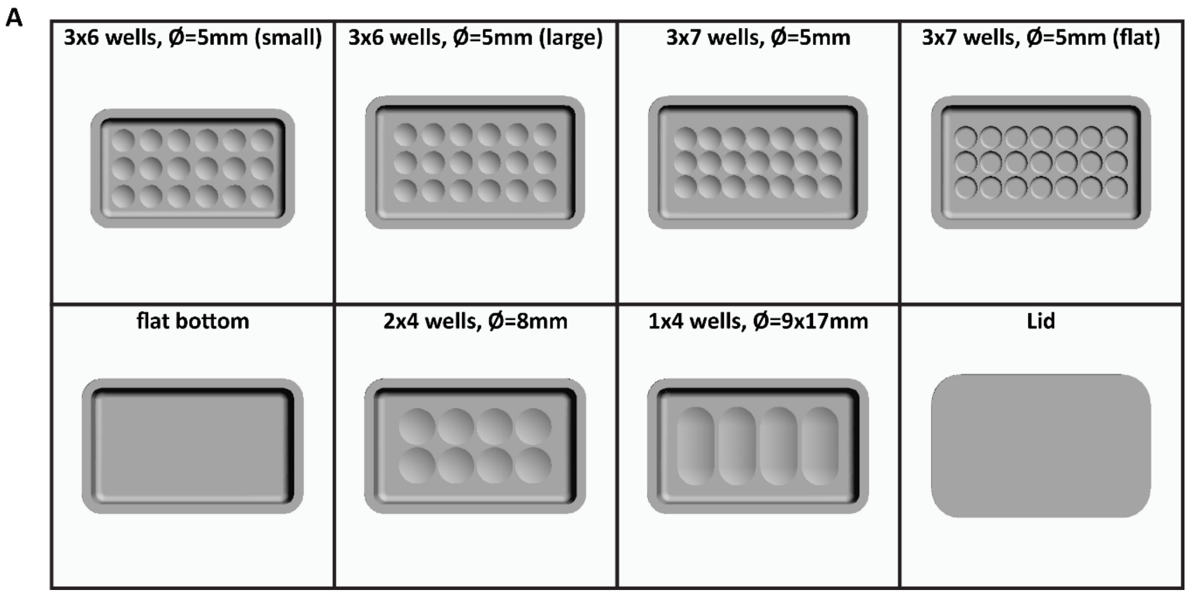

Supplemental figure 1: MTM designs. Related to figures 1-3.

A, MTM designs for various tissue sizes and numbers, adjusted for regular size microscopy slides, as well as the lid design.

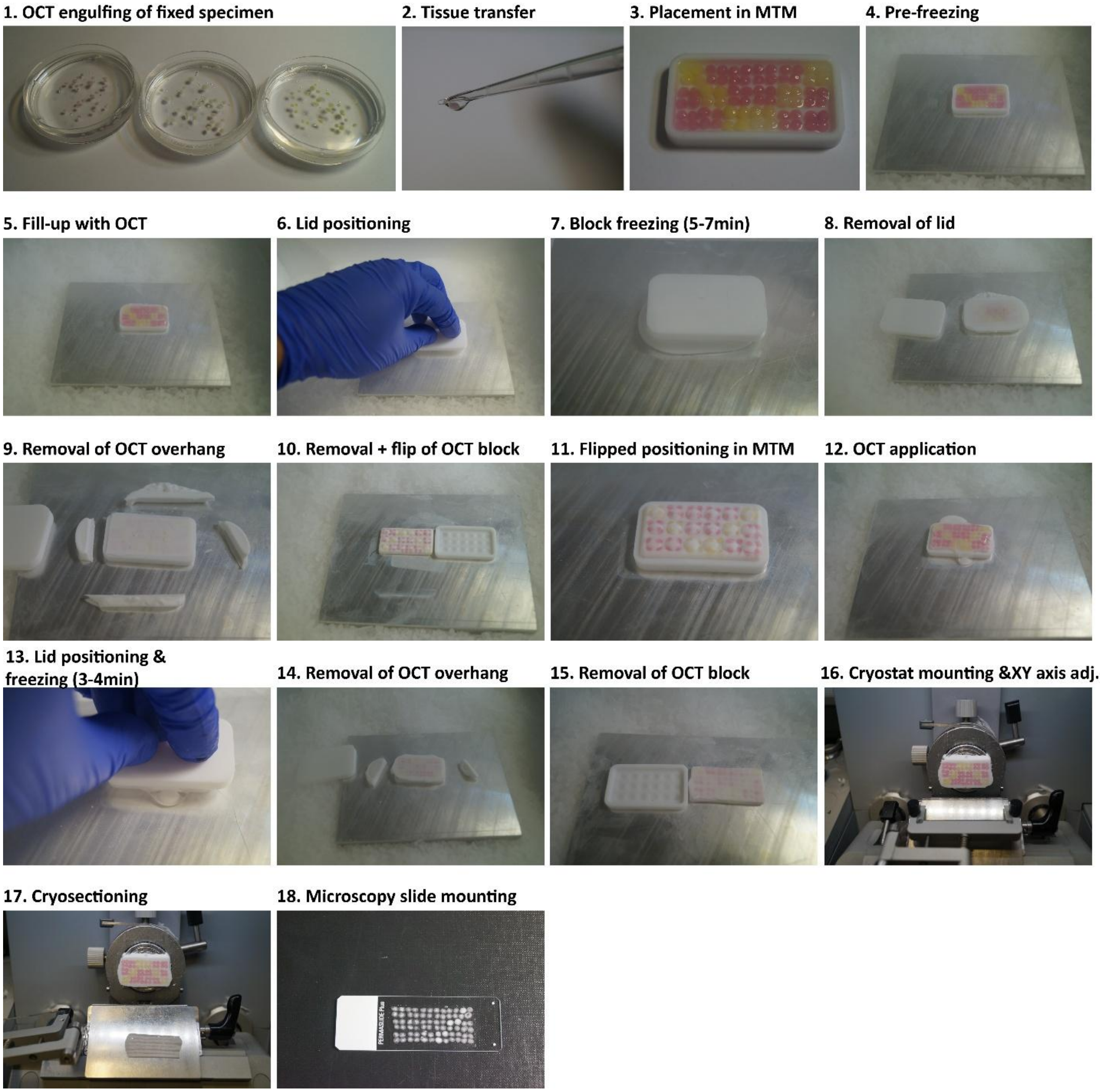

Supplemental Figure 2: Step-by-step documentation of MTM based multiplexed cerebral organoid embedding and cryosectioning. Related to figures 1-3.

Organoids expressed either GFP, tdtomato or no fluorophore (see Figure 1C, step 6).

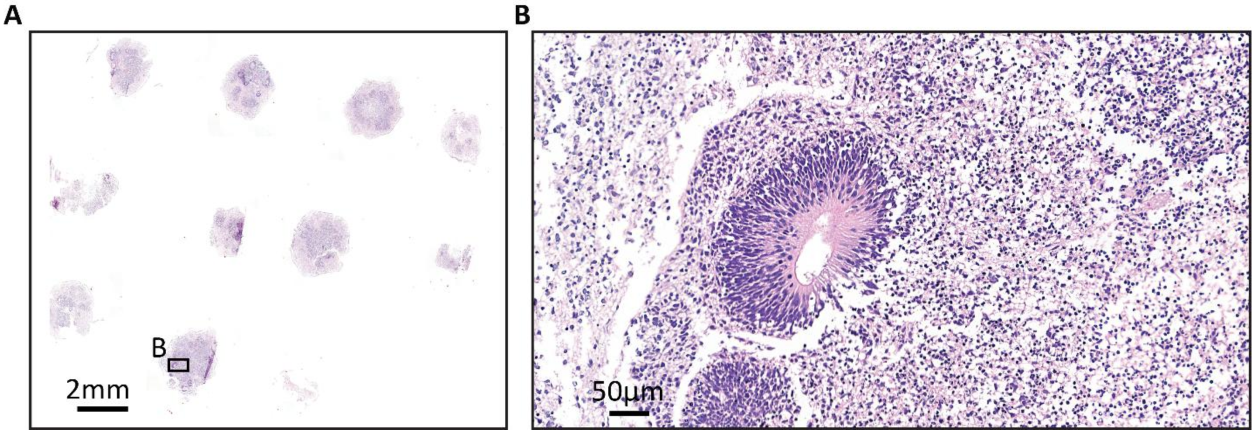

Supplemental figure 3: Paraffin embedding using MTMs. Related to Figure 1.

A, Overview of microtome sectioned organoids which were dehydrated, and paraffin embedded in MTMs (4x4 organoids with last position empty) and then sectioned on a vibratome and H&E stained. **B**, magnified view of indicated box in panel A.

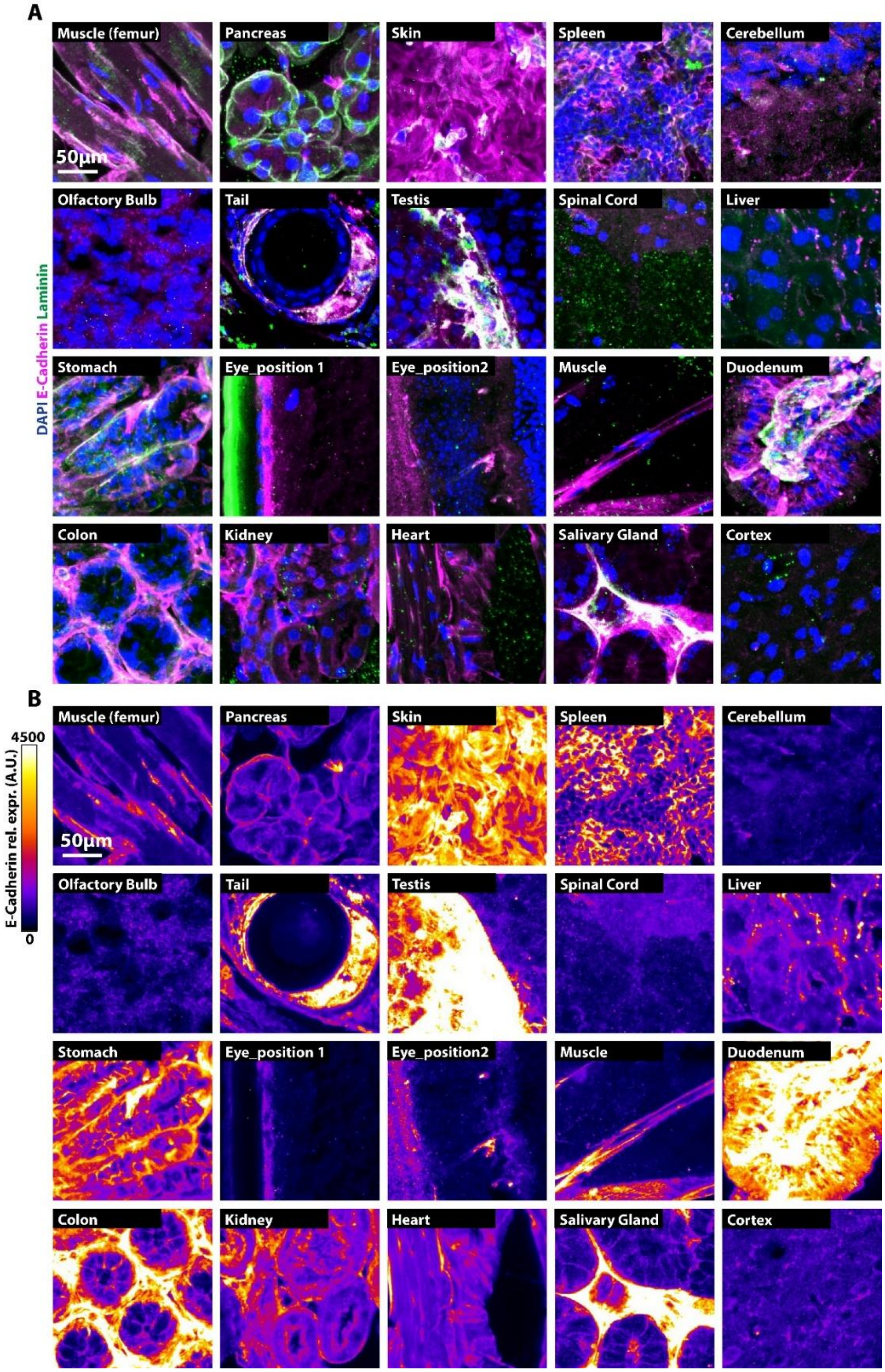

Supplemental Figure 4: High-Magnification recordings of MTM processed mouse tissues. Related to figure 2.

A, Representative 40x immunofluorescence images of MTM processed mouse organs (see also Figure 2) labeled for DAPI, E-Cadherin and Laminin. **B,** Corresponding Fire LUT representation highlighting tissue-specific E-cadherin expression levels. N= 1 mouse and 19 organs.

Supplemental Table S1: Cost list for timecourse organoid experiment. Related to Figures 1 and 3.

General Consumables			Product links	Cat No	Accessed	Price
TX100 50g (Thermo)	1.916	€/ml	https://www.thermofisher.com/order/catalog/product/85111	28313	accessed 07/01/2024	1l for 1916€
BSA (AlbuMAX) 11021037 (Thermo)	4.05	€/g	https://www.thermofisher.com/order/catalog/product/11021037?SID=srch-srp-11021037	11021037	accessed 07/01/2024	100g for 405€
PBS sterile thermo 1l 10010031	0.03282	€/ml	https://www.thermofisher.com/order/catalog/product/10010031?SID=srch-srp-10010031	10010031	accessed 07/01/2024	1l for 32,82
PBS tablets nonsterile 100 tablets (500ml each)	194	€	https://www.thermofisher.com/order/catalog/product/18912014	18912014	accessed 07/01/2024	100 tablets for 194€ (500ml each)
-->PBS tablet derived PBS	0.00388	€/ml				

Cost estimate- OCT			https://www.scienceservices.eu/tissue-tek-oct-compound.html	SA62550-12	accessed 07/01/2024	12 bottles a 125ml for 214,90€
OCT (12 bottles)	214.9	€				
per block (approx 10ml OCT):	1.517655	€				
6 wells of 6well plate (reusable), approx 24ml:	3.642373	€				
**total**	**5.160028**	€				

Cost estimate for 50ml antibody staining solution		
TX100	0.0958	€
BSA	10.125	€
PBS	0.194	€
**SUM**	**10.4148**	**€**
**SUM for 10 slides (approx. 5ml)**	**1.04148**	**€**

Cost estimate for 50ml blot/perm solution		
TX100	0.2874	€
BSA	10.125	€
PBS	0.194	€
**SUM 50ml**	**10.6064**	**€**
**SUM for 10 slides (approx. 5ml)**	**1.06064**	**€**

Cost estimate 1l PBS-T		
PBS tablet	3.88	€
TX100	0.1916	€
**SUM**	**4.0716**	€

1l PBS		
2PBS tablets (e.g. Thermo 18912014)	3.88	€
**SUM**	**3.88**	**€**

Cost estimate for 10 primary antibody IHC (200ul/staining)		
approximation:		
cost for 100µl antibody (estimated dilution: 1:300):	500	€
--> antibody costs per slide (0.67µl in 200µl AB solution):	3.35	€
**SUM (antibody cost for 10 stainings):**	**33.5**	**€**

Cost estimate for 10 secondary antibodies (fluorescently conj., 200µl/staining)		
approximation:		
per antibody for 500ul (2mg/ml conc, est. dil. 1:500):	363	€
--> antibody cost per slide (0.4µl in 200µl):	0.2904	€
**SUM (secondary antibody cost for 10 slides)**	**2.904**	**€**

Microscopy slides						
VWR 631-0108 72 slides	36.8	€	https://at.vwr.com/store/product?keyword=631-0108	631-0108	accessed 07/01/2024	36.8€ for 72 slides
**SUM (10 microscopy slides total)**	**5.111111**	**€**				

Coverslips						
VWR 24x50 631-0146 1000 pieces	152	€	https://at.vwr.com/store/product?keyword=%20631-0146	631-0146	accessed 07/01/2024	152€ for 1000 coverslips
**SUM (10 coverslips)**	**1.52**	**€**				

Mounting media						
e.g. DAKO 15ml	110	€	https://www.agilent.com/store/productDetail.jsp?catalogId=S302380-2&catId=SubCat3ECS_866	CS70330-2	accessed 07/01/2024	110€ for 15ml
SUM (for 10 slides, approx. 30µl/slide)	2.2	€				

TOTAL COST (MTM):		
**TOTAL CONSUMABLE COST (1 antibody/slide):**	**60.4489**	€
**TOTAL CONSUMABLE COST (3 antibodies/slide):**	**47.4893**	€

Cost per Analysis (approx. 114 tissues per slide (dependent on position in the block), 21 tissue groups):		
Cost per tissue (for 10 stainings, 1 staining per slide):	0.530253	€
Cost per tissue group (for 10 stainings, 1 staining/slide):	2.878517	€
Cost per tissue (for 10 stainings, 3 staining per slide):	0.416573	€
Cost per tissue group (for 10 stainings, 3 staining/slide):	2.261396	€

TOTAL COST (conventional cryomolds):						
21 groups processed separately, 10 slides for AB staining:	210	slides				
multiplexed (3 antibodies/slide, 4 slides per group):	84	slides				
Single-use cryomolds (Tissue-Tek 15x15x15, 100pc):	37.9	€	https://www.scienceservices.de/tissue-tekr-cryomoldr-kryoeinbettformen.html	SA62534-10	accessed 07/01/2024	37.9€ for 100 pc
**Total consumable cost (1 antibody/slide):**	**1349.016**	**€**				
**Total consumable cost (3 antibody/slide, 4 slides):**	**1076.866**	**€**				

Cost saving with MTM:		
**1 antibody/slide:**	**95.519**	**%**
**3 antibodies/slide, 4 slides:**	**95.59**	**%**
